# Volumineuse masse intracardiaque révélée par un accident vasculaire cérébral: à propos d’un cas

**DOI:** 10.11604/pamj.2024.48.188.42137

**Published:** 2024-08-28

**Authors:** Amngar Bekoutou, Astrid Monfort, Elysé Kisioko, Adrianna Tongavelona, Heriniaina Daddy Randriamiarisoa, Aude Aline-Fardin, Jean Philippe Lesbre, Jocelyn Inamo

**Affiliations:** 1 Service de Cardiologie Hémodynamique, Centre Hospitalier Universitaire de Martinique, Fort de France, Martinique,; 2 Centre Hospitalier Universitaire Bon Samaritain de Walia, N´Djamena, Tchad,; 3 Service de Neurologie, Centre Hospitalier Universitaire de Martinique, Fort de France, Martinique,; 4 Laboratoire d´Anatomie et Cytologie Pathologiques, Centre Hospitalier Universitaire de Martinique, Fort de France, Martinique,; 5 Département de Cardiologie, Centre Hospitalier Universitaire d´Amiens-Picardie, Amiens, France

**Keywords:** Myxome intra auriculaire, accident vasculaire cérébral ischémique, échocardiographie, cas clinique, Intra-atrial myxoma, ischemic stroke, echocardiography, case report

## Abstract

Les masses intracardiaques peuvent être révélées par un accident vasculaire cérébral ischémique. Elles sont parfois difficiles à différencier par échocardiographie. Déterminer leur nature est un élément important de la prise en charge. Nous rapportons le cas d´un homme de 58 ans, hypertendu non traité, tabagisme actif, suivi pour un psoriasis, une bronchopneumopathie chronique obstructive (BPCO) et un nodule du lobe supérieur gauche avec suspicion de cancer du poumon. Il est hospitalisé pour un accident vasculaire cérébral ischémique qui est thrombolysé avec un remaniement hémorragique. L´échocardiographie systématique révèle une volumineuse masse intra auriculaire gauche évocatrice de myxome intra auriculaire. L´analyse histologique de la pièce chirurgicale confirmera ce diagnostic. Les myxomes auriculaires gauches peuvent rester longtemps asymptomatiques et être révélés par des complications emboliques systémiques. L´échocardiographie oriente le diagnostic et l´analyse anatomopathologique le confirme. La prise en charge doit être multi disciplinaire.

## Abstract (en)

Intracardiac masses may be revealed by an ischemic cerebrovascular accident (stroke). They are sometimes difficult to differentiate by echocardiography. Determining their nature is a crucial aspect of patient management. We here report the case of a 58-year-old man with untreated hypertension, active smoking, a history of psoriasis, chronic obstructive pulmonary disease (COPD) and a nodule in the left upper lobe suspected to be lung cancer. He was hospitalized for an ischemic stroke which was treated with thrombolysis, resulting in hemorrhagic transformation. Routine echocardiography revealed a large mass in the left atrium suggestive of intra-atrial myxoma. Histological examination of the surgical specimen confirmed the diagnosis. Left atrial myxomas can remain asymptomatic for a long time and may be revealed by systemic embolic complications. Echocardiography guides the diagnosis and anatomopathological examination confirms it. Management should be multidisciplinary.

## Introduction

L'incidence des masses intracardiaques est faible. Il s´agit essentiellement de tumeurs cardiaques bénignes, de tumeurs malignes primitives ou secondaires, ou de thrombus [1-3]. Les deux tiers des tumeurs primaires sont bénignes, et plus de la moitié de ces tumeurs sont des myxomes [4]. Selon la taille et l'emplacement de la tumeur, les masses intracardiaques peuvent ne présenter aucune manifestation spécifique à l'examen physique ou des symptômes d'insuffisance cardiaque non spécifiques, notamment la dyspnée, la fatigue ou l'orthopnée. Elles peuvent être révélées par des complications thromboemboliques tels que les accidents vasculaires cérébraux [2]. L'échocardiographie est utile pour distinguer le type de masse cardiaque. La résection chirurgicale est le traitement de choix des myxomes cardiaques et l'anticoagulation est généralement recommandée pour le traitement initial des thrombi intracardiaques [2,5]. L'étude histopathologique permet de déterminer la nature de la masse. Ce cas vise à présenter les caractéristiques cliniques, para cliniques (imagerie et histologie) et la prise en charge d'une volumineuse masse cardiaque et à discuter de la nature de l´embole cérébral.

## Patient et observation

**Informations sur le patient:** monsieur B.C âgé de 58 ans, d´origine afro-caribéenne avec comme facteur de risque cardiovasculaire une hypertension artérielle non traitée et un tabagisme actif à 40 paquets par année. Il est suivi pour un psoriasis, une bronchopneumopathie chronique obstructive (BPCO) avec hippocratisme digital, et un nodule du lobe supérieur gauche faisant suspecter un cancer de poumon. Il est hospitalisé pour malaise sans perte de connaissance ni douleur thoracique.

**Résultats cliniques:** l´examen neurologique initial retrouve une hémiplégie droite avec paralysie faciale, des troubles phasiques et une désorientation temporo-spatiale avec un score NIHSS à 21.

**Figure 1:**
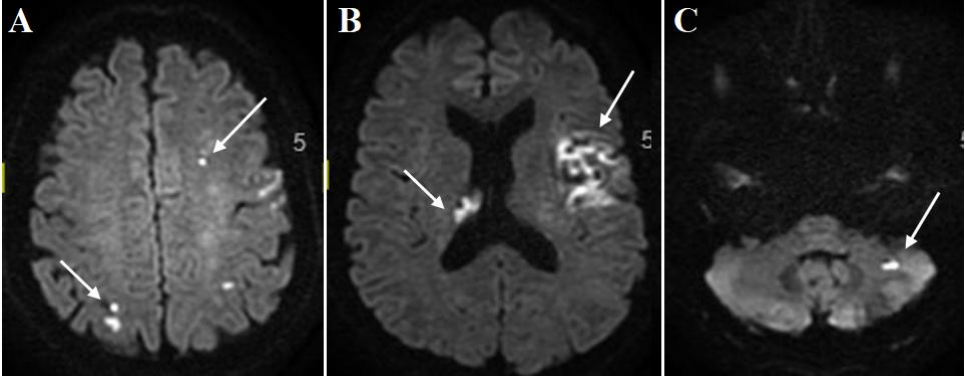
A) infarctus multiples de siège jonctionnel ACA-ACM gauche et ACP droit; B) sylvien superficiel gauche et thalamique droit; C) et cérébelleux gauche

**Figure 2:**
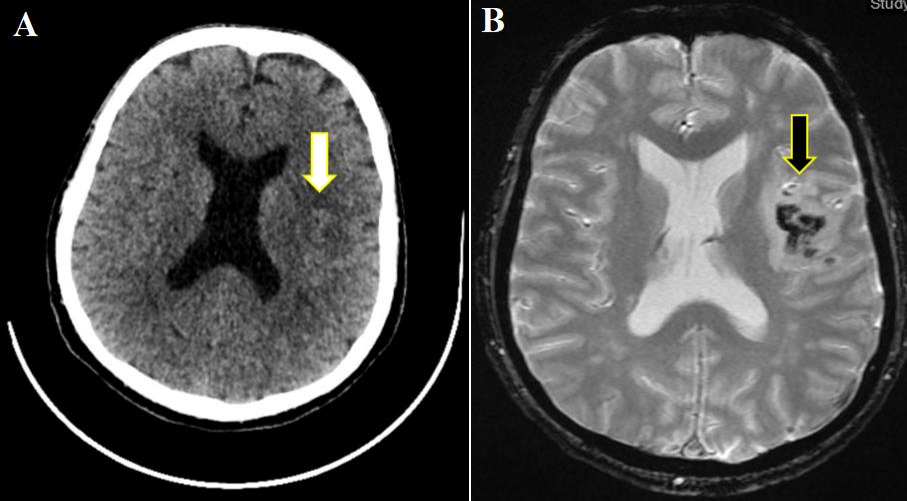
A) transformation hémorragique après thrombolyse au scanner non injecté; B) et à l'imagerie par résonance magnétique

**Évaluation diagnostique:** l´imagerie par résonance magnétique (IRM) cérébrale met en évidence des infarctus multiples dans des territoires différents: sylvien superficiel gauche avec occlusion d'une branche de trifurcation M2, jonctionnel antérieur gauche et postérieur droit (Figure 1). Il a été thrombolysé à l´Actilyse 1h50 après le début des signes, permettant une récupération neurologique avec un score NIHSS 2 (un manque du mot et une dysarthrie intelligible à la 24^e^ heure) malgré la survenue d´un remaniement hémorragique (Figure 2).

**Figure 3:**
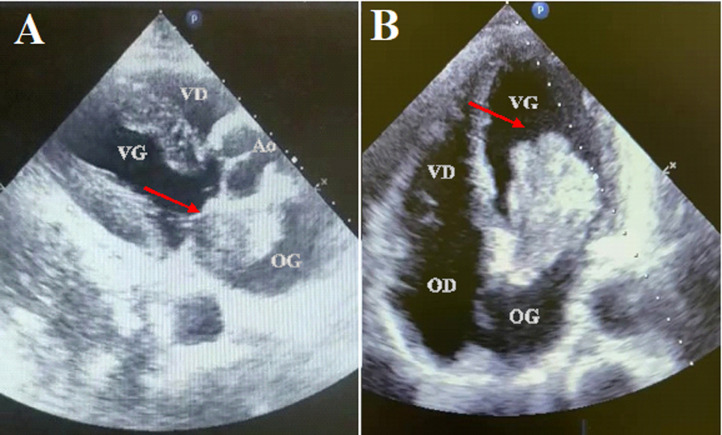
A) masse intra atriale gauche insérée sur le septum inter auriculaire; B) et faisant prolapsus dans l´orifice mitral en diastole

**Figure 4:**
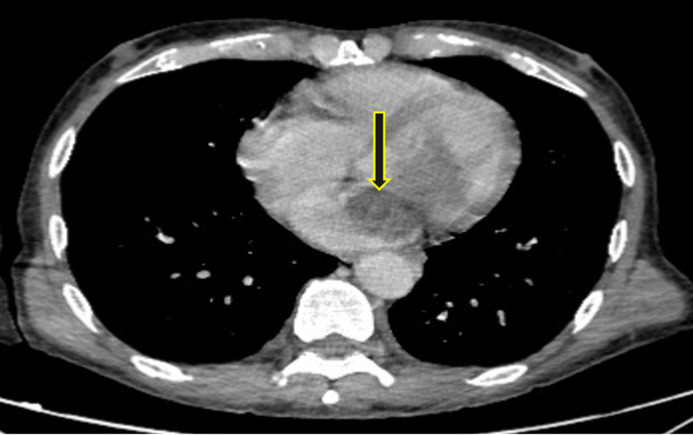
masse hypodense intra atriale gauche au scanner thoraco-abdomino-pelvien

L´échocardiographie transthoracique (ETT) retrouvait une masse intra atriale gauche (OG), sur pédicule sessile s´insérant sur le septum inter auriculaire (Figure 3). Cette masse prolabait dans l´orifice mitral en diastole, entrainant un obsatcle avec un gradient moyen OG-VG de 6 mmHg. Les autres cavités cardiaques étaient normales. L´échocardiographie trans oesophagienne (ETO) et le scanner thoraco-abdomino-pelvien trouvaient les mêmes conclusions (Figure 4). L´hypothèse d´un myxome de l´oreillette gauche a été émise.

**Figure 5:**
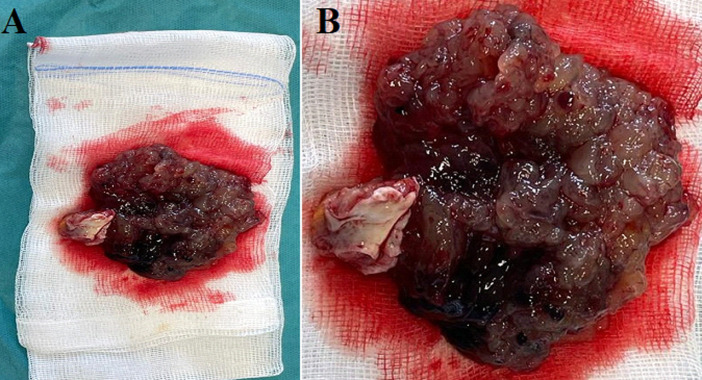
(A,B) pièce opératoire après exérèse chirurgicale

**Intervention thérapeutique:** une indication de résection chirurgicale de la masse est retenue. L´intervention est réalisée un mois plus tard pour mitiger tout risque de transformation hémorragique induite par la circulation extra-corporelle (CEC). L´intervention retrouve une volumineuse masse gélatineuse dont la base d´insertion est réséquée (Figure 5). Aucune autre lésion n´est retrouvée.

**Figure 6:**
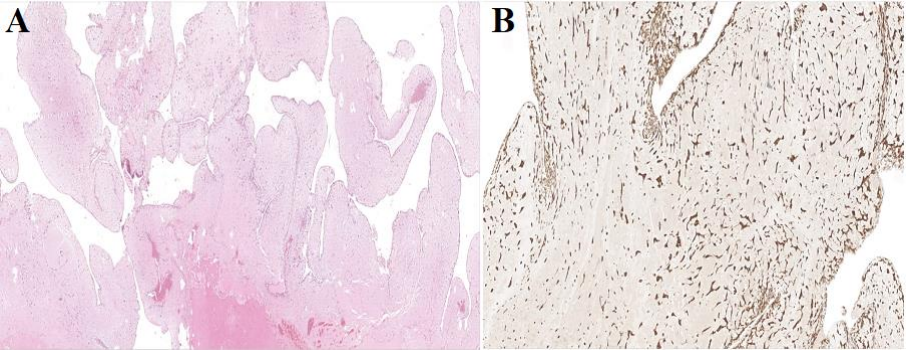
A) lésion polypoïde faiblement cellulaire avec remaniement nécrotico-hémorragique de l´axe, HES grossissement x10; B) immunohistochimie: expression de la calrétinine par les cellules ovoïdes ou stellaires

**Suivi et résultats:** les suites opératoires sont simples. L´analyse histologique confirme l´exérèse complète d´une lésion pédiculée polypoïde, faiblement cellulaire. Cette lésion est constituée de quelques cellules non atypiques, ovoïdes ou stellaires, agencées en nids ou en cordons, munies d´un noyau régulier, arrondi ou ovalaire cerné par un cytoplasme éosinophile modérèment abondant. Ces cellules se détachent sur un fond fibromyxoïde, par places dissociées par des suffusions hémorragiques et ponctuées d´un léger infiltrat inflammatoire polymorphe. La lésion comporte des vaisseaux non atypiques, assez nombreux, parfois entourés de matériel myxoïde et par les cellules précédemment décrites. Au niveau de la base d´implantation, il y avait des remaniements hémorragiques et inflammatoires. L´axe de lésion était le siège de remaniements nécrotico-hémorragiques. En immunohistochimie, le marquage à la calrétinine est positif, confirmant le diagnostic de myxome (Figure 6).

## Discussion

Le cas présenté ici illustre une des formes cliniques trompeuses d'expression des masses ou tumeurs intracardiaques. Ces masses peuvent être asymptomatiques ou se révéler par des complications telles qu´une embolie systémique. La recherche systématique d´une étiologie cardio-embolique d´un AVC était indispensable au diagnostic.

L´échocardiographie doit faire partie systématiquement des bilans étiologiques d´un AVC ischémique. Elle permet de faire le diagnostic des masses intracardiaques et de suspecter leur nature qui peut être un myxome.

Les tumeurs cardiaques primitives représentent 0,02% de l´ensemble des tumeurs. Soixante-quinze pour cent (75%) sont bénignes et sont largement dominées chez l´adulte par le myxome (50%) [2-4]. Le myxome est situé le plus fréquemment dans l´oreillette gauche (75 à 90% des myxomes) [6]. Les myxomes de l´oreillette gauche (O.G) sont des tumeurs bénignes du point de vue anatomo-pathologique mais de par leurs conséquences (embolies, enclavement dans l´orifice mitral). Ces tumeurs peuvent mettre en jeu le pronostic vital. Elles sont développées à partir de résidus embryonnaires mésenchymateux au niveau de la paroi du septum intraauriculaire. Elles se présentent sous deux aspects: la forme «gélatineuse» qui est à l´origine de complications emboliques systémiques au niveau des membres et du cerveau et la forme «fibreuse» arrondie et ferme qui est à l´origine des enclavements dans l´orifice mitral.

Dans notre cas clinique, devant l´antécédent du nodule du lobe supérieur gauche faisant suspecter un cancer de poumon, l´hypothèse d´une tumeur secondaire pourrait être évoquée. Mais la localisation intra auriculaire gauche de la masse et l´absence de foramen ovale persistant ont fait évoquer l´hypothèse d´un myxome. Après l´exérèse chirurgicale, l´analyse histologique de la masse intraauriculaire gauche était en faveur d´un myxome. Ce dernier était de forme «fibreuse» arrondie et ferme. Alors que c´est la forme gélatineuse qui est à l´origine de complications emboliques systémiques. Toutefois, au niveau de la base d´implantation de ce myxome de forme «fibreuse» arrondie et ferme, il y avait des remaniements hémorragiques et inflammatoires. L´axe de la lésion était le siège de remaniements nécrotico-hémorragiques qui pourraient former des emboles. Ainsi l´AVC ischémique de notre patient serait dû à des emboles formés secondairement aux remaniements nécrotico-hémorragiques.

Vu que c´est seulement le myxome de forme «gélatineuse» qui est à l´origine de complications emboliques systémiques, la problématique de thrombolyse dans les AVC ischémiques sur un myxome intraauriculaire se pose. Le scanner cérébral et surtout l´IRM cérébrale pourront préciser la nature de l´embole cérébral.

## Conclusion

Le myxome demeure une cause rare d´AVC ischémique. Ce cas souligne l´importance d´un bilan cardiaque systématique dans le bilan étiologique des AVC ischémiques en particulier quand ils touchent plusieurs territoires. L´échocardiographie transthoracique constitue l´examen clé dans le dépistage des masses intra cardiaques.
